# Responses of germination strategy of *Agriophyllum squarrosum* to rainfall pattern in the Tengger desert

**DOI:** 10.7717/peerj.14395

**Published:** 2022-11-15

**Authors:** Meiling Liu, Ruiqing Zhu, Huichun Xie

**Affiliations:** 1 Qinghai Provincial Key Laboratory of Medicinal Plant and Animal Resources of Qinghai-Tibet Plateau, College of Life Science, Qinghai Normal University, Xining, Qinghai, China; 2 Academy of Plateau Science and Sustainability, Qinghai Normal University, Xining, Qinghai, China

**Keywords:** *Agriophyllum squarrosum*, Germination, Dormancy, Species dynamic, Rainfall pattern

## Abstract

*Agriophyllum squarrosum* (L.) Moq. (Chenopodiaceae) is an annual pioneer psammophyte that is strictly distributed along desert margins. However, little is known about how this species adapts to shifting dunes. In this study, seeds bank was selected and germination behaviors of *A. squarrosum* were tested in laboratory. In addition, the effects of rainfall patterns on population dynamics were observed in field at the southeastern edge of the Tengger Desert. Soil seed bank density was significantly different in different depth of sand dunes. Under adequate water in Petri dishes, seeds began to germinate in less than 3 h and the germination peak was reached in seven days after watering. It showed that there is no innate dormancy of *A. squarrosum*. The buried experiments showed that the germination percentage decreased with increasing buried depth, and deeply buried seeds (10 cm) remained ungerminated. Population dynamics in different rainfall pattern of three years in field showed that germination, survival and deaths of *A. squarrosum* were extremely sensitive to rainfall variation. Our results suggest that precipitation is the key factor in determining population of *A. squarrosum.* The germination strategy of *A. squarrosum* ensures the efficiency use of unpredicted and scarce precipitation. The high disturbance of moving sand endowed persistence seed bank of *A. squarrosum,* which is essential for population continuation, avoiding population extinction under unpredicted precipitation.

## Introduction

Shifting sand dunes are characterized by low water-holding capacities, extreme temperatures, low nutrient levels, and unstable substrate (Olsson & Wilhelmsson, 2000). Thus, very few psammophytes can survive in this harsh desert habitat (Ma & Liu, 2008). *Agriophyllum squarrosum* (L.) Moq. is among the plants that successfully grow in this harsh habitat. *A. squarrosum* is an annual pioneer psammophyte that grows nearly exclusively on shifting sand dunes and is widely distributed over the arid zones of China, Russia, Mongolia, and Iran (Liu et al., 2007). *A. squarrosum* is believed to be conducive to sand stabilization (Zheng et al., 2004). With the restoration of sand dunes, *A. squarrosum* will be replaced by other psammophytes. The local name of *A. squarrosum* in Western China is “sand rice.” This species has a high concentration of nutrients in its seeds and other organs, representing a new crop alternative for future food production (Chen et al., 2014). Given its high ecological and economic values, *A. squarrosum* has recently attracted increasing attention in studies of physiology traits, soil seed banks, community dynamics, and transcriptomic analysis (Bai, Bao & Li, 2004; Zhang et al., 2005; Liu et al., 2006; Zhao et al., 2014). The root system of *A. squarrosum* has a rather unique structure and includes a long taproot and nearly equally long lateral roots near the soil surface (Chen et al., 2014). These characteristics enable the plant to gain a foothold in sand and withstand strong sandstorms. A set of genes that are likely relevant for resistance to heat stress were functionally annotated in *A. squarrosum* (Zhao et al., 2014)*.* The physiology and morphology of sand rice are ideally adapted to extreme desert conditions (Bai, Bao & Li, 2004), but how the mechanisms of *A. squarrosum* are exclusively adapted to shifting sand dunes remains unclear.

Seed dormancy and germination are critical elements of the plant life cycle, and information on these issues of *A. squarrosum* is important in understanding the temporal and spatial dynamics of this species in shifting sand dunes (Commander et al., 2017). The precipitation in arid desert is characterized by unpredictability in temporal dynamics and amount. Germination occurs when the precipitation reaches five mm (Zhang et al., 2001). However, the seedlings will die in the absence of precipitation after germination. If all the seeds are germinated simultaneously, then population extinction may occur under unpredicted environments. Dormancy is crucial in the formation of soil seed banks, and long-lived seed banks maintained by annual desert plants are often regarded as evolutionary bet-hedging strategies against unpredictable environmental variations (Gutiérrez & Meserve, 2003). Germination experiments have been conducted on the seeds of *A. squarrosum* (Zheng et al., 2004; Li et al., 2006a; Li et al., 2006b; Liu et al., 2013), but the results on whether the seeds of *A. squarrosum* have a dormancy characteristic remain inconsistent. The seed germination of *A. squarrosum* exhibits a unimodal continuous pattern, reaching the highest percentage at seven days after sowing in the field (Zheng et al., 2004). Li et al. (2006) found that fresh seeds show rapid germination with a high percentage (>90%), whereas the germination percentage of stored seeds decreases significantly. In the research of Liu et al. (2013), dry storage under laboratory conditions has limited effects on the germination of *A. squarrosum*. Little is known about the adaptive seed germination strategies of *A. squarrosum* in temperate desert, which is characterized by unpredicted precipitation. The information gap constrains our understanding of the population continuation mechanism of this species.

In this study, the specific objectives are to investigate the seed bank distribution and the germination of *A. squarrosum* to ascertain whether it has dormancy and understand its population continuation mechanism in shifting sand dunes. Therefore, we first investigated the seed bank distribution in different types of sand-dunes. Then, we conducted germination experiments on the optimum watering regime and different burial depths to examine whether dormancy exists in *A. squarrosum*. Furthermore, the germination dynamics in the field under different rainfall patterns was observed to verify previous dormancy results. Our main objective here is to test the hypothesis that the high disturbance of shifting is a mechanism that endows seed dormancy to preserve the seed bank of *A. squarrosum* in shifting dunes. This test will elucidate the mechanism that enables *A. squarrosum* to grow nearly exclusively on shifting sand dunes, providing information for vegetation management in arid and semi-arid deserts and guidance for our subsequent domestication of this plant and agricultural practice to cope with future food security.

## Methods and Materials

### Botanical description

The species is an annual psammophyte, which grows to a height of 20–100 cm, forming erect, obscurely ribbed stems, which branch from the base (Chen et al., 2014). The ripening season of *A. squarrosum* is from September to March of the second year. The seeds stored in the canopy were significantly more than dispersal. More than half of the seeds are released from the plant to the soil between March and May (Gao et al., 2014). The seeds of *A. squarrosum* are small and mostly oval in shape. For the seed size, the average major axis of the seeds is 1.96 mm, ranging from 1.17 mm to 2.99 mm. For the seed weight, the average thousands of seed weight is 1.33 g (Zhao et al., 2022).

### Study sites

This study was conducted at the Shapotou Desert Experimental Research Station on the southeast fringe of the Tengger Desert, China (37°27′N, 104°57′E, 1330 m a.s.l). The area is characterized by high, dense, and continuous reticulate barchan dunes. The soil substrate is loose with impoverished shifting sand and 2%–3% moisture. The annual mean precipitation and temperature are 186 mm and 10 °C, respectively (Liu et al., 2016). The precipitation is characterized by high uncertainty and the only water source in this area (Wang et al., 2019).

### Seedling germination traits in controlled experiments

Seeds were collected on November 6, 2015. The seeds were randomly collected from both large and small plants and in different sections of the sand dunes. The seeds were mixed together and stored in a cool dry room at 4 °C.

Two experiments of seedling germination were conducted in this study: (1) germination under proper conditions, and (2) germination in different soil depths. Experiment (1) was conducted immediately after the seeds were collected, and three replicates of 50 seeds were placed on moistened filter paper in 15-cm Petri dishes. The dishes were placed in a greenhouse (16/8 h photoperiod; 25 °C/12 °C day/night; PAR 150 mol/m^2^/s, relative humidity 30%). Germination was considered with radicals protruding at least one mm through the seed coat. Once germination began, seedlings were counted and removed every day; meanwhile, deionized water was added to the Petri dishes to ensure adequate moisture. Counting continued until no additional germination was observed over a period of seven days. The seeds that had not germinated by this time were removed from the Petri dishes and checked for viability using the method of MacKay (1972). The ungerminated seeds’ coats were removed, and the embryos were soaked in 1% tetrazolium chloride at 30 °C for two days. Pink embryos were tagged as alive. The germination percentage was calculated by dividing the number of viable seeds by the number of germinated seeds per day.

To simulate germination in the field, germination experiments (2) were carried out in Shapotou desert and the experimental station. Germinations were conducted in pots of 20 cm diameter and 25 cm height containing soil from the collection site. Seeds were planted in 2-cm increments at depths ranging from the surface to 20 cm to test the germination percentage. Fifty seeds were sown at each depth with three replicates. The pots were then placed in the field without shield, except for rainy days. The experiments started on July 1, 2016. All the pots were watered sufficiently twice every day. The counting and calculation methods were as described previously in experiment (1).

### Seed bank sampling and analysis

Seed banks were sampled in March of 2016. For active sand dunes, three quadrats, from the bottom of the windward slope to the bottom of the leeward slope, were set with 30-m intervals. In each quadrat, four cores (20 cm × 20 cm) were collected at 10-m intervals. For each core, the sampling depths were 0–20, 20–40, 40–60, 60–80, and 80–100 cm. The seeds of *A. squarrosum* were isolated at different soil depths. The seed viability was determined by the tetrazole staining method, and the number of viable seeds was counted and calculated (seeds/m^2^) at different soil depths.

### Species dynamics in different rainfall pattern

**Figure 1:**
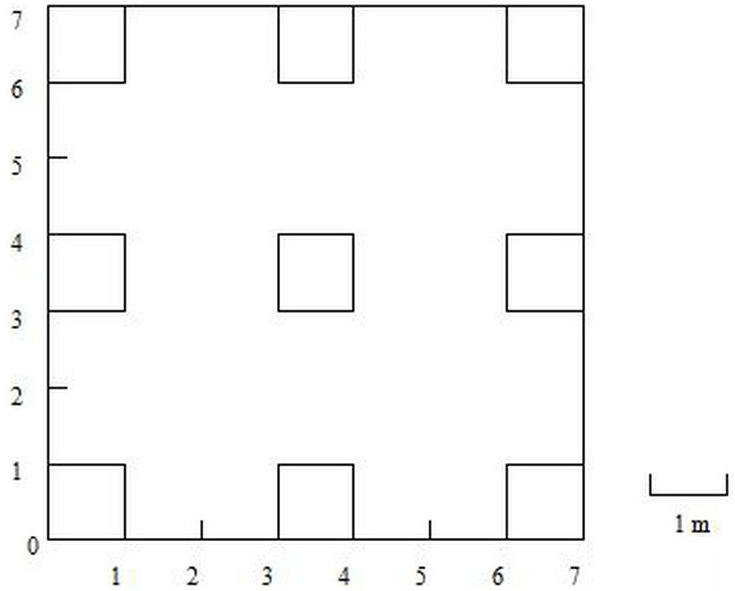
Sketch of sample plots.

The rainfall was measured with a tipping bucket rain recorder at the Shapotou meteorological station. On the shifting sand dunes, a 7 m × 7 m transect was established and divided into 9 1 m × 1 m quadrats with an interval of 2 m to survey the species dynamics of *A. squarrosum* (Fig. 1). The observation was performed from April to November of 2017 to 2019. All the germinated seedlings were labeled with numbers and then identified when grown up. The death of *A. squarrosum* was also recorded. The density was calculated as the number of living *A. squarrosum* per m^2^.

### Statistical analysis

Each experiment was performed at least in triplicate, and all data are presented as means ± standard deviation. The data were analyzed and processed using Microsoft Excel version 2010. Multiple comparisons of the means were performed through the least significant difference test.

## Results

### Germination traits of *A. squarrosum* seeds

**Figure 2:**
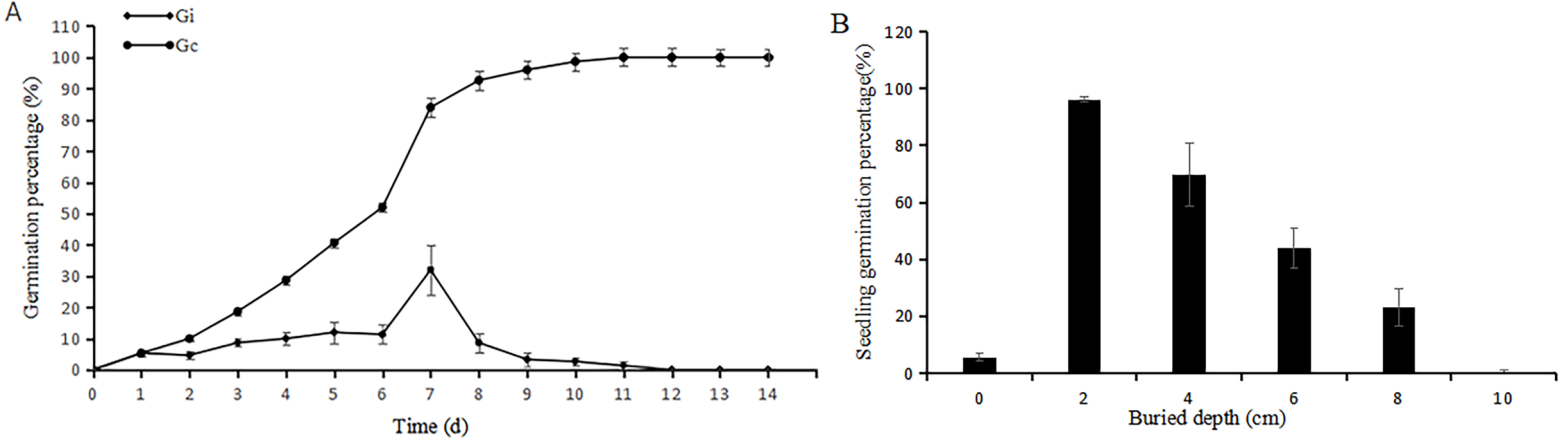
Germination percentage in Petri dishes under adequate water (A) and in soil at different buried depth (B). Gi (*i* = 1, 2, …, 14) represents germination percentage of a given day; Gc (Cumulative germination percentage) represents total germination percentage of a given day. Data were presented as means ± SD.

In germination experiment (1), the seeds began to germinate less than three hours after watering (Fig. 2A). Continuous moisture exposure had a significant effect on the germination percentage, which reached the peak on day 7. All the seeds germinated after 14 days, and the seed germination percentage reached 100%. The cumulative germination percentage of the first seven days was 68.8%, and the germination percentage of the seventh day was 32.8%.

In buried germination experiment (2), the germination percentage of the seeds on the surface is low (6.3 ± 1.8%) (Fig. 2B). The germination percentage of the seeds at 2-cm depth increased significantly and nearly completely geminated. Then, the germination percentage decreased with the increasing buried depth. The deeply buried seeds (10 cm) remained ungerminated.

### Vertical distribution traits of seed bank on active sand dunes

**Figure 3:**
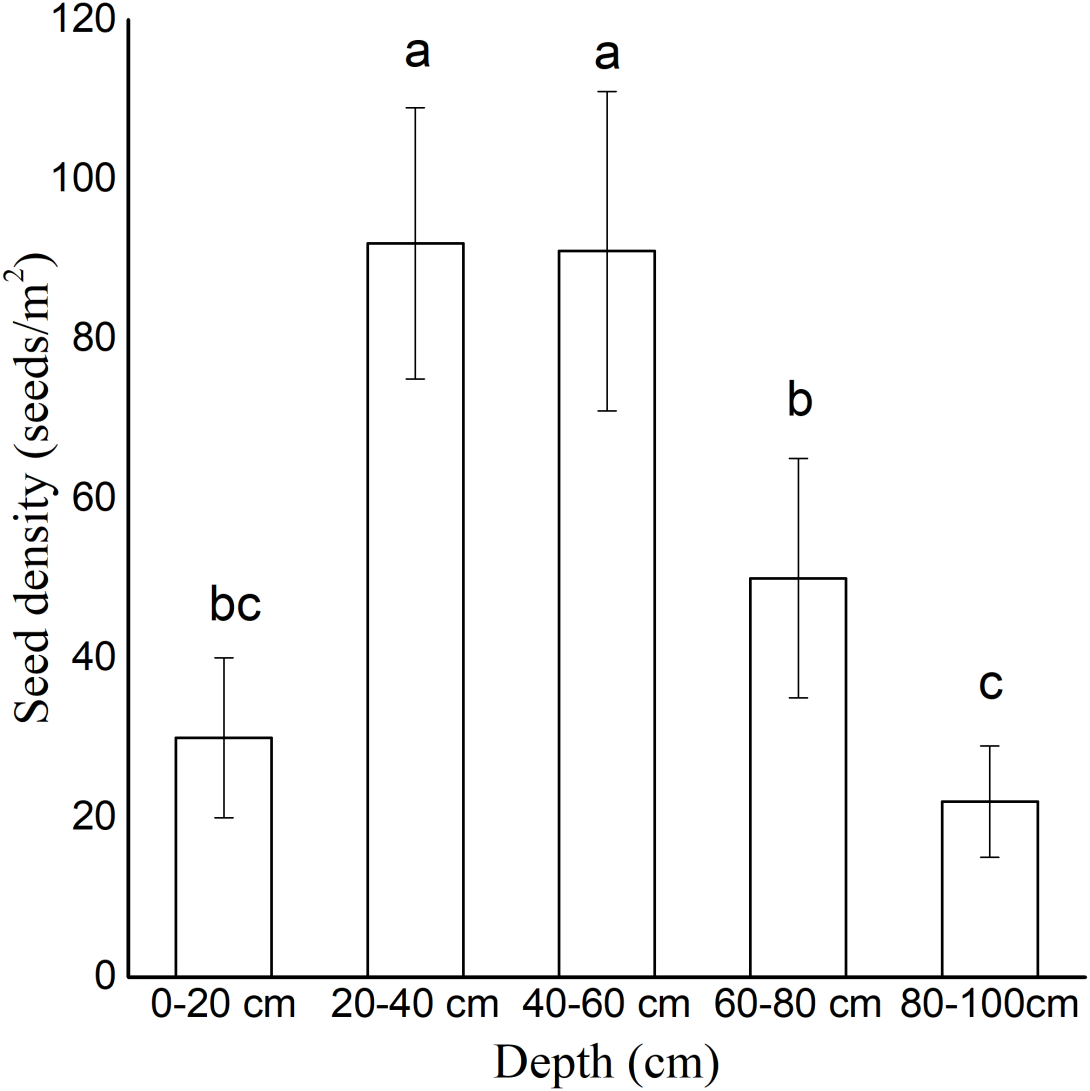
Density of persistent seed bank at different depths. All sampling points were included. Data were presented as means ± SD.

The soil seed bank density varied significantly at different sand dune depths. The seeds were vertically concentrated in the depths of 20–40 cm (93 ± 23 seeds/m^2^) and 40–60 cm (88 ± 14 seeds/m^2^) (Fig. 3). The seeds in the 60–80 cm depth (50 ± 15 seeds/m^2^) were fewer than those in the other depths. In the depths of 0–20 cm and 80–100 cm, the density of the seeds was less than 30 seeds/m^2^.

### Species dynamics in different rainfall pattern

**Figure 4:**
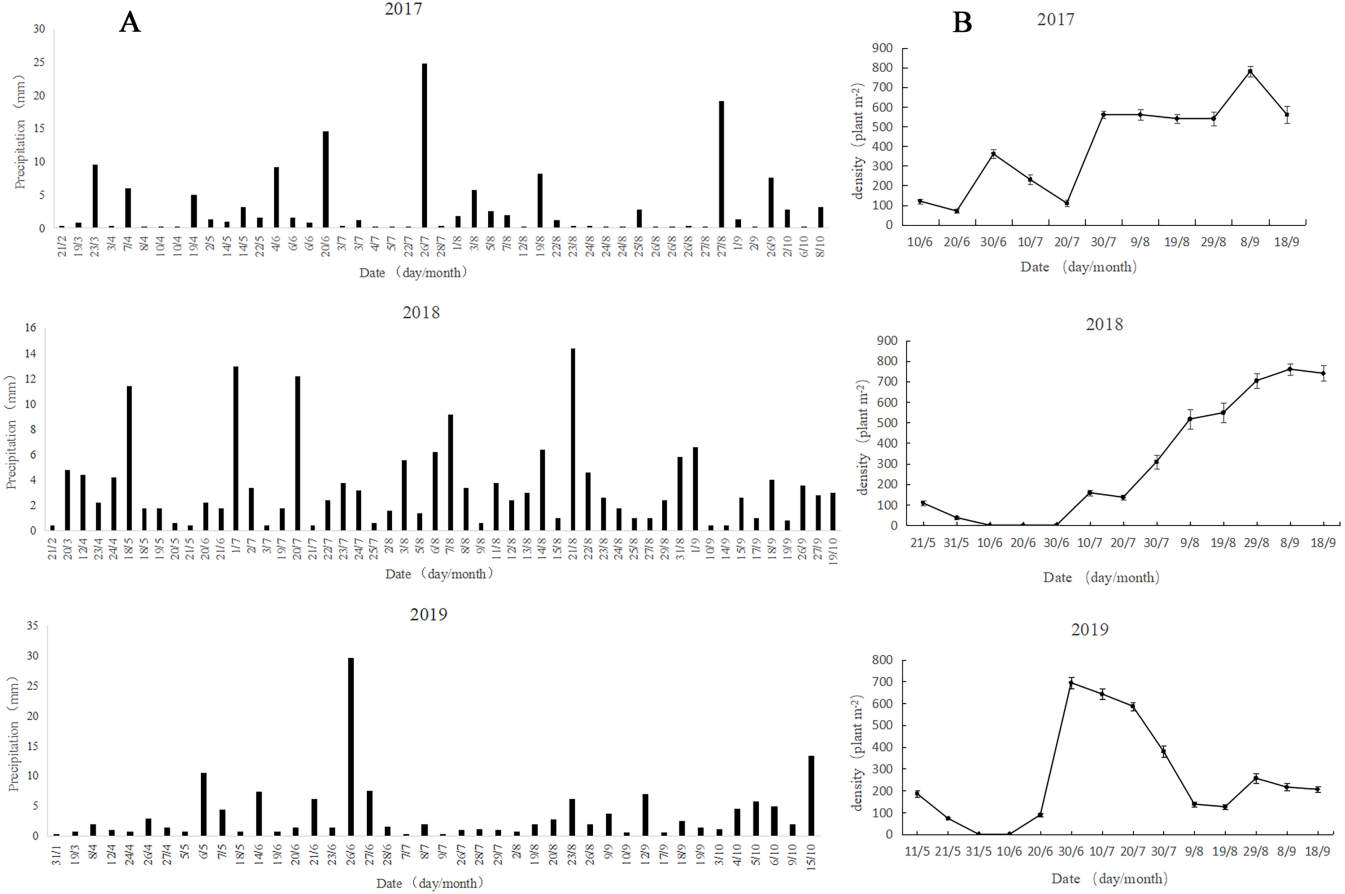
Patterns of rainfall (A) and dynamics of *A. squarrosum* (B) during 2017, 2018 and 2019.

The precipitation in 2017 and 2018 was mainly concentrated in July and August, and the precipitation in 2018 was stable and sustainable (Fig. 4A). In 2019, the precipitation showed significant variability, with precipitation concentration in May and June, followed by nearly no precipitation from July to August, while strong precipitation occurred in October. The overall precipitation showed strong randomness and uncertainty.

The population dynamics in 2017 showed that the germination of *A. squarrosum* was in batch (Fig. 4B). The first group of seedlings emerged from the precipitation on June 4, but some of them withered on June 20 because of the absence of precipitation support. The heavy rainfall (14.6 mm) on June 20 helped with the survival of the first surviving batch of seedlings and nursed the germination of the second group of *A. squarrosum*. However, a long period of drought followed, resulting in the death of many seedlings, especially for some newly sprouted seedlings. More precipitation was experienced in August (46 mm), and the third batch of seedlings emerged and survived successfully, exhibiting continuity. The emergence of a fourth batch of seedlings was also observed. The population dynamics in 2018 were significantly different from those in 2017. Seed banks germinated immediately in response to precipitation, and the subsequent continuous and stable precipitation pattern maintained the survival and growth of seedlings. The population of *A. squarrosum* continued to increase steadily, and no large numbers of seedlings perished. The large soil seed bank capacity and the stable seedling survival rate kept the relative stability of population characteristics. The precipitation in 2019 showed extreme uncertainty. The population dynamics of *A. squarrosum* also showed great fluctuation. The occurrence of heavy precipitation on May 6 (10.6 mm) and June 14 (7.4 mm) respectively nursed the seeds to germinate immediately. However, no effective precipitation replenishment occurred in the later stage, resulting in the large amount of withering of the previously emerged seedlings. Only a few seedlings that survived completed their life cycle.

## Discussion

The establishment of annual plant species on active dunes is constrained by seed availability, seedling emergence, and seedling survival (Liu et al., 2006). In our germination experiment (1), seeds began to germinate less than three hours after watering. The seed germination percentage reached 100% in 14 days under continuous moisture exposure, indicating that the seeds of *A. squarrosum* have no innate dormancy in sufficient water conditions. In the buried germination experiment, the germination percentage decreased with the increasing buried depth. The deeply buried seeds (10 cm) remained ungerminated. The buried depth endowed dormancy traits on the seeds of *A. squarrosum*. The investigation of the seed bank on moving sand dunes showed that the seeds were vertically concentrated in the depth of 20–40 cm (Fig. 3). Chen et al. (2020) showed that the fixation of sand dunes has great influence on the soil seed bank. The density of the soil seed bank in the dune was higher than that in the semi-fixed dune, and nearly no seed bank was found in the fixed dune. We hypothesized that the deeply buried seeds may result in a persistent seed bank. The deeply buried seeds remained ungerminated but viable even when the soil water content was suitable for germination (Tobe, Zhang & Omasa, 2005). A persistent seed bank ensures the sustainable supply of viable seeds, thus contributing to seedling recruitment in disturbed environments (Urban, 2005). Therefore, we presume that wind erosion and sand burial result in the vertical distribution of the seed bank, which is favorable for seed bank conservation. The high disturbance of moving sand dunes helps *A. squarrosum* accumulate its seed banks and thus adapt to the environment, which is characterized by unpredictable precipitation.

The observation of the germination, growth, and death in response to rainfall pattern changes in the field is critical to verifying the germination in laboratory and understanding the adaptive mechanisms of plant population under altered climate regimes. Desert annuals respond quite rapidly to rainfall changes throughout their entire life cycle and show different responses to unpredictable precipitation (Chen et al., 2019). In this study, the precipitation pattern showed great viability in occurrence time and amount in 2017, 2018, and 2019 (Fig. 4A). Meanwhile, the species germination, growth, survival, and death showed significantly different responses to different precipitation patterns (Fig. 4B). The population dynamics in 2017 showed that the germination of *A. squarrosum* was in batches. A portion of seedlings perished during the short-term absence of rainfall. Thus, precipitation seems the key factor of *A. squarrosum* seedling survival in our study. The population dynamics in 2018 were significantly different from those in 2017. Seed banks germinated immediately in response to precipitation, and the continuous and steady precipitation pattern maintained the survival and growth of seedlings. No large seedling deaths occurred, and the population of *A. squarrosum* continued to increase steadily. The precipitation in 2019 showed extreme uncertainty. The population dynamics of *A. squarrosum* also showed great fluctuation. The occurrence of a large amount of precipitation encouraged the seeds to germinate rapidly. However, a long period of absence of effective precipitation replenishment occurred in the later stage, which led to a large number of deaths of the previously emerged seedlings. Only a few surviving seedlings completed their life cycle. Similar results have been observed in the rainfall manipulation experiments on annual plants (Karimmojeni et al., 2014; Wang et al., 2019; Gao et al., 2015). These results indicate that *A. squarrosum* shows strong sensitivity in response to rainfall variation, which enhances its ability to recruitment in the unpredictable environments of active sand dunes.

Therefore, the seed germination strategy is characterized by swift response and high germination rate, which can be classified as the opportunistic germination strategy. The population dynamics in the field also show that the germination, growth, survival, and death are greatly dependent on precipitation. This strategy can ensure rapid germination and settlement after precipitation. However, the germination strategy can cause the extinction of population under highly unpredictable water regimes. Interestingly, the seeds buried at different soil depths showed different germination percentages, and the spatial heterogeneity led to dormancy for *A. squarrosum*. This phenomenon can prevent the simultaneous germination of all the seeds, forming a persistence seed bank, which can effectively avoid population extinction when little water is available in the following time. Overall, the germination strategy of *A. squarrosum* ensures the efficient use of unpredicted and scarce precipitation. The high disturbance of moving sand endows the seed bank of *A. squarrosum* persistence, which is essential for population continuation, avoiding population extinction under unpredictable precipitation.

##  Supplemental Information

10.7717/peerj.14395/supp-1 Supplemental Information 1 Raw data for figures

## Additional Information and Declarations

### Data Availability

The following information was supplied regarding data availability: The raw measurements are available as a Supplemental File.

## References

[ref-1] Bai WM, Bao XM, Li LH (2004). Effect of *Agriophyllum squarrosum* seed banks on its colonization in a moving sand dune in Hunshandake Sand Land of China. Journal of Arid Environments.

[ref-2] Chen F, Ma QL, Wei LY, Zhang DK, Yuan HB, Ding F, Hu XK, Zhang Z (2020). Soil seed bank characteristics of *Agriophyllum squarrosum*. Journal of Desert Research.

[ref-3] Chen GX, Zhao JC, Zhao X, Zhao P, Duan R, Nevo E, Ma X (2014). A psammophyte *Agriophyllum squarrosum* (L.) Moq.: a potential food crop. Genetic Resources and Crop Evolution.

[ref-4] Chen Y, Zhang L, Shi X, Liu H, Zhang D (2019). Life history responses of two ephemeral plant species to increased precipitation and nitrogen in the Gurbantunggut Desert. PeerJ.

[ref-5] Commander LE, Golos PJ, Miller BP, Merritt DJ (2017). Seed germination traits of desert perennials. Plant Ecology.

[ref-6] Gao R, Yang X, Liu G, Huang Z, Walck JL (2015). Effects of rainfall pattern on the growth and fecundity of a dominant dune annual in a semi-arid ecosystem. Plant and Soil.

[ref-7] Gao R, Yang X, Yang F, Wei L, Huang Z, Walck JL (2014). Aerial and soil seed banks enable populations of an annual species to cope with an unpredictable dune ecosystem. Annals of Botany.

[ref-8] Gutiérrez JR, Meserve PL (2003). El Niño effects on soil seed bank dynamics in north-central Chile. Oecologia.

[ref-9] Karimmojeni H, Bazrafshan AH, Majidi MM, Torabian S, Rashidi B (2014). Effect of maternal nitrogen and drought stress on seed dormancy and germinability of Amaranthus retroflexus. Plant Species Biology.

[ref-10] Li XH, Jiang DM, Liu ZM, Li XL (2006b). Seed germination characteristics of annual species in temperate semi-arid region [in Chinese]. Acta Ecologica Sinica.

[ref-11] Li XR, Xiao HL, He MZ, Zhang JG (2006a). Sand barriers of straw checkerboards for habitat restoration in extremely arid desert regions. Ecological Engineering.

[ref-12] Liu ZM, Yan QL, Baskin CC, Ma J (2006). Burial of canopy-stored seeds in the annual psammophyte *Agriophyllum squarrosum* Moq. (Chenopodiaceae) and its ecological significance. Plant and Soil.

[ref-13] Liu HL, Zhang LW, Yin LK, Zhang D-Y (2013). Effects of temperature, dry storage, and burial on dormancy and germination of seeds of 13 desert plant species from sand dunes in the Gurbantunggut Desert, Northwest China. Arid Land Research and Management.

[ref-14] Liu ML, Zhu RQ, Zhang ZS, Liu L, Hui R, Bao J, Zhang H (2016). Water use traits and survival mechanisms of psammophytes in arid ecosystems. Arid Land Research and Management.

[ref-15] Liu Z, Yan Q, Liu B, Ma J, Luo Y (2007). Persistent soil seed bank in *Agriophyllum squarrosum* (Chenopodiaceae) in a deep sand profile: variation along a transect of an active sand dune. Journal of Arid Environments.

[ref-16] Ma JL, Liu ZM (2008). Spatiotemporal pattern of seed bank in the annual Psammophyte *Agriophyllum squarrosum* Moq. (Chenopodiaceae) on the active and dunes of northeastern inner Mongolia, China. Plant and Soil.

[ref-17] MacKay DB, Roberts EH (1972). The measurement of viability. Viability of seeds.

[ref-18] Olsson PA, Wilhelmsson P (2000). The growth of external AM fungal mycelium in sand dunes and in experimental systems. Plant and Soil.

[ref-19] Tobe K, Zhang L, Omasa K (2005). Seed germination and seedling emergence of three annuals growing on desert sand dunes in China. Annuals of Botany.

[ref-20] Urban KE (2005). Plant species dynamics during restoration of heath ponds in northwestern Germany. Phytocoenologia.

[ref-21] Wang Y, Li X, Liu L, Zhao J, Sun J (2019). Life history response of *Echinops gmelinii* Turcz. to variation in the rainfall pattern in a temperate desert. PeerJ.

[ref-22] Zhang JG, Li XR, Wang XP, Wang G (2001). Population dynamic of annual plant *Eragrostis poaeoides* in fixed sand dune in Shapotou area. Journal of Desert Research.

[ref-23] Zhang J, Zhao H, Zhang T, Zhao X, Drake S (2005). Community succession along a chronosequence of vegetation restoration on sand dunes in Horqin Sandy Land. Journal of Arid Environments.

[ref-24] Zhao PS, Capella-Gutiérrez S, Shi Y, Zhao X, Chen G, Gabaldón T, Ma X-F (2014). Transcriptomic analysis of a psammophyte food crop, sand rice (*Agriophyllum squarrosum*) and identification of candidate genes essential for sand dune adaptation. BMC Genomics.

[ref-25] Zhao PS, Li XF, Ran RL, Sun H, Zhao J, Chen G (2022). Precipitation and local environment shape the geographic variation of seed size across sand rice (*Agriophyllum squarrosum*) natural populations. Journal of Experimental Botany.

[ref-26] Zheng YR, Gao Y, An P, Shimizu H, Rimmington GM (2004). Germination characteristics of *Agriophyllum squarrosum*. Canadian Journal of Botany.

